# Retraction: Mechanisms of hybrid oligomer formation in the pathogenesis of combined Alzheimer’s and Parkinson’s diseases

**DOI:** 10.1371/journal.pone.0321152

**Published:** 2025-03-18

**Authors:** The PLOS One Editors

The *PLOS One* Editors retract this article [1] following concerns about the reliability and integrity of the results reported in Figs 1 and 5. Specifically,

- In the Fig 1A α-syn panel, there appear to be irregularities in the background surrounding the 14kDa band in lane 3.
- The following lanes appear more similar than would be expected from independent results:
  - Fig 1A Aβ (6E10) panel, lanes 5-6 and lanes 7-8
  - Fig 1D α-syn panel, lane 5 and lane 6
- There appears to be a vertical irregularity in the background of the Fig 1A Aβ (6E10) panel, between lanes 2 and 3.

The corresponding author provided data underlying the image results presented in Figs 1, 5, and 6. These did not resolve the concerns raised with the Fig 1A Aβ (6E10) panel and the Fig 1D α-syn panel. In addition, discrepancies observed between the published Fig 5A and the underlying data provided for this figure raised further concerns about the integrity of the published Fig 5A, which call into question the article’s findings that solubilized Aβ and Aggregated Aβ induces aggregation of α-syn into the formation of stable dimers and trimers.

The following additional issues were resolved upon follow-up:

- Lanes 4-12 of the Fig 1D Actin panel in this article [1] appear similar to lanes 4-12 of the Fig 9C Actin panel of [2] despite being used to represent different experimental conditions. The underlying blot data confirmed that the Actin panel presented in Fig 1D of [1] is correct.
- Vertical irregularities were observed between lanes 6-7 in Fig 5A and 5B, and between lanes 3-4 in Fig 5A and 5D. Underlying data confirm that Fig 5A–5D were prepared using spliced results from non-adjacent lanes of the same underlying blot, resulting in the appearance of vertical irregularities in the background of these panels.
- The underlying data provided for Fig 6D shows that an area of this panel was enlarged in the published figure and there appears to be a vertical irregularity within the panel. However, PLOS considers that the underlying data provided support the reported results.

IFT, BS, and EM did not agree with the retraction. BS stands by the published results. LC, PD, GMS, YS, HM, ER, MT, OP, and JXJY either did not respond directly or could not be reached.

## References

[1] Tsigelny IF, Crews L, Desplats P, Shaked GM, Sharikov Y, Mizuno H, et al. Mechanisms of hybrid oligomer formation in the pathogenesis of combined Alzheimer’s and Parkinson’s diseases. PLoS ONE. 2008;3(9): e3135. doi: 10.1371/journal.pone.0003135 18769546 PMC2519786

[2] Overk CR, Cartier A, Shaked G, Rockenstein E, Ubhi K, Spencer B, et al. Hippocampal neuronal cells that accumulate α-synuclein fragments are more vulnerable to Aβ oligomer toxicity via mGluR5--implications for dementia with Lewy bodies. Mol Neurodegener. 2014;9: 18. doi: 10.1186/1750-1326-9-18 24885390 PMC4041038

